# Validation of the Chinese version of the adaptive functions of music listening scale among Chinese university students

**DOI:** 10.3389/fpsyg.2026.1881426

**Published:** 2026-10-05

**Authors:** Xin Lyu, Bin Pan, Liang Chen, Linqin Ji, Wenxin Zhang

**Affiliations:** 1 Faculty of Psychology, Shandong Normal University, Jinan, China; 2 Shandong Provincial Key Laboratory of Brain Science and Mental Health, Jinan, China

**Keywords:** adaptive functions of music listening, measurement invariance, reliability, subjective well-being, validity

## Abstract

**Background:**

A multidimensional measure of music-listening functions is needed in the Chinese context. This study evaluated the psychometric properties of the Adaptive Functions of Music Listening Scale (AFML) among Chinese university students.

**Methods:**

A total of 2,829 university students were recruited from Weifang Institute of Technology in Shandong, China. Confirmatory factor analysis (CFA), measurement invariance testing, reliability analysis, and criterion validity analysis were conducted.

**Results:**

CFAs supported the original 11-factor structure of the AFML. Multi-group confirmatory factor analyses indicated that this structure was invariant across gender and musical training experience. The subscales demonstrated good to excellent internal consistency. Regression analyses further showed that several adaptive AFML dimensions were associated with better well-being, whereas rumination showed a consistent maladaptive pattern across affective and social indicators. In addition, identity was associated with higher positive affect and life satisfaction, but also with higher negative affect.

**Conclusion:**

The Chinese version of the AFML demonstrated good reliability and validity, supporting its use for assessing the adaptive functions of music listening among Chinese university students.

## Validation of the Chinese version of the adaptive functions of music listening scale among Chinese university students

Music listening is one of the most common and accessible forms of engagement with music. The widespread use of mobile devices and streaming platforms has made music listening an integral part of everyday life (Feneberg et al., 2026). On average, individuals aged 16–64 worldwide spend 20.7 h per week listening to music (International Federation of the Phonographic Industry, 2023). Music listening is more than entertainment; it serves multiple adaptive functions in everyday life (Feneberg et al., 2026). Previous conceptual work has described these functions as involving affective, social, eudaimonic, and goal-related purposes, including emotion regulation, social connection, meaning, and personal growth (Groarke and Hogan, 2016). These functions may vary across individuals and contexts and should not be treated as interchangeable. Understanding these functions is important for clarifying how individuals use music in daily life and how music listening may be associated with psychological well-being. The diversity of these functions also highlights the need for multidimensional instruments that can assess music listening comprehensively. Researchers have developed a range of self-report instruments to assess multiple music-related functions and experiences (Koehler et al., 2026). However, existing measures typically capture only specific aspects of music listening (e.g., emotion regulation, Chin and Rickard, 2012). Moreover, the extent to which the conceptual frameworks and psychometric properties of these measures apply to the Chinese context has yet to be established. In the present study, we examined the psychometric properties of a multidimensional measure—the Adaptive Functions of Music Listening Scale—among Chinese university students.

### The adaptive functions of music listening scale

Research on music-listening functions has largely relied on self-report instruments, which mainly capture individuals’ subjective experiences and uses of music in daily life (Groarke and Hogan, 2018). For example, the Barcelona Music Reward Questionnaire focuses on reward-related experiences in music listening (Mas-Herrero et al., 2013), the Music Use Questionnaire assesses active engagement with music across regulatory, social, and activity-based domains (Chin and Rickard, 2012), and the Motives for Listening to Music Questionnaire examines listening motives related to enhancement, coping, social, and conformity purposes (Kuntsche et al., 2016). Taken together, these instruments assess related but distinct aspects of music listening, including individuals’ motives and uses of music, as well as their subjective emotional, cognitive, and social experiences during listening. However, a validated multidimensional measure that comprehensively assesses a broad range of music-listening functions in Chinese populations has not yet been established.

The Adaptive Functions of Music Listening Scale (AFML, Groarke and Hogan, 2018) is a self-report measure designed to assess the functions that individuals perceive music listening to serve. In this context, adaptive functions of music listening refer to the perceived purposes or expected outcomes associated with listening to music, such as regulating affect, supporting cognitive engagement, maintaining social connectedness, and addressing everyday psychological needs. Notably, the term “adaptive” does not imply that all AFML dimensions are uniformly beneficial or that music listening invariably produces positive outcomes across individuals or contexts. The original scale comprises 46 items representing 11 dimensions of music-listening functions, namely *Stress Regulation, Anxiety Regulation, Anger Regulation, Loneliness Regulation, Rumination, Reminiscence, Strong Emotional Experiences, Awe and Appreciation, Cognitive Regulation, Identity*, and *Sleep*. These functions broadly reflect affective, social, and cognitive domains of music listening. Specifically, the scale covers affective experiences and affect regulation, social connectedness and identity-related functions, as well as cognitive functions related to concentration, reflection, and appreciation. The original scale demonstrated a clear multidimensional structure and acceptable psychometric properties, making it a useful foundation for further refinement and validation.

The AFML was developed and validated in Western samples. It remains unclear whether its conceptual structure and psychometric properties can be generalized to non-Western cultural contexts. Prior research suggests that music serves several broadly similar functions across cultures, including mood regulation, social bonding, and identity expression (Boer et al., 2012). However, the social functions of music listening, particularly its role in family and peer rituals and social cohesion, may vary in salience and cultural embeddedness across settings (Boer and Abubakar, 2014). This issue is particularly relevant in the Chinese context characterized by collectivism that emphasizes the maintenance of interpersonal harmony, compliance with authority, and conformity to group norms (Triandis, 2001). In the Chinese cultural context, music-listening functions may be more closely tied to socially embedded experiences. Therefore, it is important to examine whether the AFML can adequately capture music-listening functions in this context.

### Functions of music listening by gender and musical training experience

Establishing measurement invariance is a prerequisite for meaningful comparisons of music-listening functions across groups. Due to potential differences in the nature and meaning of music use by gender and musical training experience, it is important to test the measurement invariance of the AFML across these groups. Regarding gender, women listeners have been reported to show stronger music-related emotional and social responses, including emotion evocation, mood regulation, and social reward (Mas-Herrero et al., 2013; Wang et al., 2023). Thus, gender differences may be particularly evident in the emotional and social functions of music.

Music training not only enhances auditory perception and music information processing but also shapes how individuals process emotional information in musical contexts (Che et al., 2026). Recent experimental research with musically trained Chinese participants has shown that musical esthetic emotion processing involves distinguishable components, including esthetic attitudes, esthetic judgments, and emotional experiences (He et al., 2025). These findings provide further evidence that engagement with music among musically trained listeners involves both affective and cognitive components. Individuals with formal music training tend to exhibit more sensitive and polarized valence responses during music listening (Heng et al., 2026). Moreover, longer musical training experience may lead individuals to place greater emphasis on both emotional and cognitive uses of music. For individuals with music training experience, music often serves as a source of communication, self-expression, emotionality, and creative flow (Woody and McPherson, 2010). Therefore, musical training experience may be associated with stronger endorsement of the emotional, cognitive, and communicative functions of music.

### The association between functions of music and well-being

From the perspective of social cognitive theory (Bandura, 2001), beliefs and expectations about the outcomes of music listening are formed through direct experience and vicarious learning. These beliefs may shape how listeners attend to, interpret, and subjectively experience musical activities (Bandura, 1986). Positive beliefs about music listening may further encourage active engagement in musical activities, and successful listening-related experiences may in turn strengthen these beliefs (Groarke and Hogan, 2018). Therefore, beliefs about the adaptive functions of music listening may promote individuals’ well-being. Subjective well-being refers to individuals’ cognitive evaluations of their lives and their affective experiences, typically encompassing life satisfaction, positive affect, and negative affect (Diener, 1984). Life satisfaction represents the cognitive component, whereas positive affect and negative affect represent affective components. Perceived social support reflects an individual’s subjective perception and evaluation of the extent to which they are supported by external sources (Zimet et al., 1988) and has been moderately associated with subjective well-being (Chu et al., 2010). To fully capture the affective, cognitive, and social correlates of music listening, positive affect, and negative affect, life satisfaction, and perceived social support were included as criterion variables.

Among the various functions of music use, stress and emotion regulation are the most commonly reported (Cook et al., 2019). Music can induce positive affective states (Fountain et al., 2026) and facilitate coping with negative affective states (Papinczak et al., 2015). Individuals who use music for stress and emotion regulation tend to report lower levels of negative affect and higher levels of positive affect and well-being (Chin and Rickard, 2012; Groarke and Hogan, 2018). However, the emotional consequences of music listening are not uniformly positive. Music use involving rumination or reminiscence has been associated with higher negative affect (Chin and Rickard, 2012; Groarke and Hogan, 2018), and some forms of music listening have been associated with psychological distress (Carlson et al., 2015). For example, strong emotional experiences during music listening, characterized by intense, deep, diverse, and mixed emotions, were associated with both positive and negative affect (Groarke and Hogan, 2018). In addition, music-induced reminiscence and rumination were linked to higher negative affect (Chin and Rickard, 2012; Groarke and Hogan, 2018). Notably, music-evoked sadness is not necessarily experienced as purely aversive; under certain conditions, it may provide esthetic, reflective, and emotional value (Taruffi and Koelsch, 2014).

Cognitive functions represent another important domain of music listening. The AFML includes two dimensions relevant to this domain, namely cognitive regulation and awe and appreciation (Groarke and Hogan, 2018). Prior research suggests that music can be used to regulate cognitive states such as focus and attention (Chamorro-Premuzic and Furnham, 2007; Chin and Rickard, 2012). By facilitating concentration and sustaining goal-directed engagement, music may support the attainment of everyday academic and personal goals, which are closely tied to emotional well-being and broader positive functioning (Groarke and Hogan, 2018; Schultheiss et al., 2008). In addition, reflective engagement with music may evoke awe and appreciation and promote insight and self-reflection (Groarke and Hogan, 2018), which may further contribute to psychological well-being (Ryff and Singer, 2008). However, Groarke and Hogan (2018) found that awe and appreciation was positively associated with negative affect. Given the mixed findings, such associations need further exploration.

Music is often linked to social bonding (Herff et al., 2025; Boer et al., 2012). The AFML includes two socially relevant dimensions, namely identity and loneliness regulation (Groarke and Hogan, 2018). During emerging adulthood, individuals actively develop their self-concept, seek a place in society, and often experience heightened feelings of loneliness and social uncertainty (Arnett, 2000). In this context, music may support identity development and expression by providing a resource for self-exploration, meaning-seeking, and the communication of personal values, preferences, and group affiliations (Myrie et al., 2022). Music-related identity functions may promote self-acceptance and personal growth, thereby improving individual psychological well-being (Groarke and Hogan, 2018). Furthermore, as a shared activity, music can provide a basis for communication, mutual enjoyment, and the formation of collective identities (Boer and Abubakar, 2014). Even in solitary listening contexts, music may help alleviate loneliness by providing a sense of companionship, emotional comfort, and symbolic connectedness (Herff et al., 2025). Through these processes, music may reduce feelings of loneliness, strengthen interpersonal ties, and enhance perceived social support (Feneberg et al., 2026; Silverman, 2014).

Sleep support is another important function of music use. Music is widely used as a self-help strategy to facilitate sleep. A substantial proportion of listeners report using music at bedtime to help them fall asleep (Trahan et al., 2018). Research has shown that music-based sleep interventions can improve subjective sleep quality and reduce sleep-onset latency (Hu et al., 2023) and may also alleviate anxiety and depressive symptoms (Zhao et al., 2024). As better sleep is closely linked to more positive affect, less negative affect, and higher well-being, individuals who use music as a sleep aid may experience emotional benefits indirectly through improved sleep.

### The current study

Despite a growing body of research emphasizing the important role of music in predicting psychosocial adjustment (Dingle et al., 2021; Miranda, 2021), there remains a lack of validated multidimensional instruments for assessing music-listening functions in the Chinese cultural context. In the present study, we aimed to validate the Chinese version of the AFML. First, confirmatory factor analyses (CFAs) were conducted to examine its initial construct validity. Second, measurement invariance and mean-level differences across gender and music-training experience (i.e., 3 years or more vs. less than 3 years) were tested. Based on previous study (Mas-Herrero et al., 2013; Wang et al., 2023; Che et al., 2026; Woody and McPherson, 2010), We hypothesized that women and students with more music training experience would score higher on the emotional, social, cognitive, and communicative AFML dimensions. As direct evidence for dimensions such as Sleep, Rumination, Reminiscence, and Loneliness Regulation is lacking, comparisons involving these dimensions were treated as exploratory. Third, the internal consistency of each AFML dimension was evaluated using Cronbach’s α and split-half reliability. Finally, based on previous findings (Groarke and Hogan, 2018; Chin and Rickard, 2014; Garrido and Schubert, 2013; Saarikallio et al., 2013; Koehler et al., 2023), criterion validity was examined by testing the associations of the 11 AFML dimensions with positive affect, negative affect, life satisfaction, and perceived social support.

We examined the psychometric properties of the AFML in a sample of first-year and second-year university students. This is a particularly relevant developmental stage for validating the AFML for two reasons. First, music listening is highly prevalent during emerging adulthood (International Federation of the Phonographic Industry, 2023) and is commonly used by university students in everyday life for emotional, cognitive, and stress-related purposes (Julia et al., 2025). Second, emerging adulthood is a critical developmental period marked by identity exploration, instability, and increasing psychosocial demands (Arnett, 2000), which may heighten the salience of music as a resource for emotion regulation, self-expression, and adjustment (Layland et al., 2018).

## Methods

### Participants and procedure

Participants were 2,829 university students recruited from Weifang Institute of Technology in Shandong, China, including 1,454 first-year students (54.0% women, *M*_age_ = 18.85 years, SD = 1.07; 46.0% men, *M*_age_ = 18.84 years, *SD* = 0.97) and 1,375 s-year students (59.8% women, *M*_age_ = 19.81 years, *SD* = 1.05; 40.2% men, *M*_age_ = 19.99 years, *SD* = 0.94). Nearly all the participants were of Han ethnicity (which constitutes 91.11% of the population in China; National Bureau of Statistics of China, 2021) and native Mandarin speakers (the language spoken by over 80% of the population; National Language Commission of China, 2022). In this sample, 69.3% majored in humanities and social sciences, and 30.6% majored in science and engineering.

Prior to data collection, researchers sent consent letters to students, teachers, and school principals briefly describing the research aims and procedures. The students involved were offered a gift (about $1). Through these procedures, 96.8% of the students contacted for the study provided active consent to participate. Students completed self-report questionnaires in a 45-min class session in the computer rooms. They were instructed to complete the questionnaires independently and were not permitted to communicate with one another during the assessment. The research design and procedure were reviewed and approved by the Human Subjects Research Ethics Committee in the Ethical Committee of Shandong Normal University (Reference Number: SDNU2025099).

### Translation process

With permission from the original authors, the Adaptive Functions of Music Listening Scale (AFML; Groarke and Hogan, 2018) was translated into Chinese using the translation–back-translation procedure. Two bilingual psychology researchers who were native Chinese speakers independently translated the English version into Chinese. They then synthesized their translations into a preliminary Chinese version of the AFML by resolving discrepancies in wording and meaning. Second, another translator who was fluent in both Chinese and English and blind to the original English version back-translated the preliminary Chinese version into English. Third, the original English version and the back-translated version were compared by two bilingual psychology professors with expertise in developmental psychology. Given that the AFML was intended for use with university students, these professors evaluated the developmental appropriateness, semantic and conceptual equivalence, cultural comprehensibility, and clarity of the items, instructions, and response options. Any discrepancies were discussed and resolved by consensus, and revisions were made when necessary, resulting in a pre-final Chinese version of the AFML. Finally, 10 university students (4 women and 6 men; age range = 18–21 years, M = 19.60, SD = 0.97; 2 first-year and 8 s-year students) completed the pre-final Chinese scale and participated in individual interviews to assess the clarity of the instructions, the comprehensibility of the items, and the appropriateness of the response format. Based on their feedback, minor wording adjustments were made to ensure that all items were clear and unambiguous.

### Measures

Demographic variables. Participants reported their gender and the total number of years they had received formal instruction in singing and/or playing a musical instrument. Accordingly, music training duration was operationalized as the cumulative number of years of formal vocal and/or instrumental training. For the analyses, gender was coded as 0 = woman and 1 = man. Music training duration was dichotomized as 0 = less than 3 years and 1 = 3 years or more.

Original Chinese Version of the Adaptive Functions of Music Listening Scale (AFML). The AFML was originally developed by Groarke and Hogan (2018). The scale includes 11 dimensions, including *Stress Regulation* (4 items), *Anxiety Regulation* (7 items), *Anger Regulation* (7 items), *Loneliness Regulation* (3 items), *Rumination* (4 items), *Reminiscence* (4 items), *Strong Emotional Experiences* (6 items), *Awe and Appreciation* (3 items), *Cognitive Regulation* (2 items), *Identity* (4 items), and *Sleep* (2 items). Item 17 and Item 37 were negatively worded and was reverse-scored before analysis. Participants rated all 46 items on a 5-point Likert scale (1 = Strongly Disagree to 5 = Strongly Agree). Two negatively worded items—one in the Reminiscence subscale and one in the Awe and Appreciation subscale—were reverse-scored. For each of the 11 dimensions, a subscale score was calculated by averaging the scores of its constituent items. Higher scores indicated stronger endorsement of the belief that music listening served the corresponding function.

Satisfaction with life. The Satisfaction with Life Scale (SWLS; Diener, 1984) comprises five items (e.g., “The conditions of my life are excellent.”), each rated on a 7-point scale from 1 (*strongly disagree*) to 7 (*strongly agree*), with higher scores indicating greater life satisfaction. Cronbach’s α for the SWLS was 0.882.

Positive and negative affect. The positive and Negative Affect Schedule (PANAS; Watson et al., 1988) was used to assess Positive Affect and Negative Affect. The scale consists of 10 Positive Affect items (e.g., “*interested*” and “*active*”) and 10 Negative Affect items (e.g., “*afraid*” and “*guilty*”). Participants were asked to indicate the extent to which they had experienced each emotion during the previous 2 weeks on a 5-point Likert scale ranging from 1 (very slightly or not at all) to 5 (extremely). Higher scores on the positive- and negative-affect subscales indicated higher levels of positive and negative affect, respectively. The Cronbach’s α coefficients were 0.953 for positive affect and 0.941 for negative affect.

Social support. Perceived social support was assessed using the Brief Form of Perceived Social Support Questionnaire (F-SozU K-6; Kliem et al., 2015). The six items were rated on a 5-point Likert scale ranging from 1 (does not apply) to 5 (exactly applicable). Item scores were averaged to obtain an overall perceived social support score, with higher scores reflecting higher levels of perceived social support. Cronbach’s α for the score was 0.917.

### Statistical analysis

Data were analyzed using SPSS 26.0 and Mplus 8.3. First, the full sample (*n* = 2,829) was randomly divided into a calibration sample (sample 1: *n* = 1,414) and a cross-validation sample (sample 2: *n* = 1,415). No significant differences were observed between the two subsamples in terms of gender (χ^2^(1) = 1.82, *p* = 0.177), age (*t*(2827) = 0.10, *p* = 0.917), and musical training experience (χ^2^(1) = 0.14, *p* = 0.711). A confirmatory factor analysis (CFA) was conducted in sample 1 to examine the hypothesized 11-factor structure of the Chinese Adaptive Functions of Music Listening Scale (AFML). Each item was specified to load on its corresponding latent factor, and cross-loadings were fixed to zero. Maximum likelihood estimation was used. Model fit was evaluated using the comparative fit index (CFI), Tucker–Lewis index (TLI), root-mean-square error of approximation (RMSEA) with its 90% confidence interval (90% CI), and standardized root mean square residual (SRMR). CFI and TLI values above 0.90 and RMSEA and SRMR values below 0.08 were considered indicative of acceptable model fit (Little, 2013). Standardized factor loadings below 0.40 were considered for item removal (Stevens, 2002). Next, a CFA was conducted in sample 2 to cross-validate the revised 11-factor model. The same model specification was applied without further data-driven modifications. This split-sample procedure was used to reduce the risk of sample-specific overfitting and to evaluate the stability and generalizability of the factor structure across independent subsamples.

Second, measurement invariance across gender and music training were tested. Following the procedure recommended by Meredith and Teresi (2006), the baseline model was first examined separately for women and men. as well as for groups with less than 3 years and 3 years or more of musical training, to ensure adequate fit of the established factor structure in each group. Next, configural, metric (weak), and scalar (strong) invariance were tested by comparing a series of nested models with increasingly stringent cross-group equality constraints. Besides the above cut-off values of the model fit indices (CFI and TLI ≥ 0.90, RMSEA and SRMR ≤ 0.08), changes in CFI, RMSEA, and SRMR were also considered, following the recommendations of Chen (2007). For metric invariance, a change in CFI of no more than 0.01, accompanied by a change in RMSEA of no more than 0.015 or a change in SRMR of no more than 0.03, was considered acceptable. For scalar invariance, a change in CFI of no more than 0.01, together with a change in RMSEA of no more than 0.015 or a change in SRMR of no more than 0.01, was considered acceptable. After establishing measurement invariance, mean-level gender differences and music training differences in adaptive functions of music listening were examined.

Third, Cronbach’s α coefficients and split-half reliability coefficients were calculated for each dimension of the AFML to assess internal consistency. Values of 0.70 to 0.79 were considered acceptable, values of 0.80 to 0.89 were considered good, and values of 0.90 or higher were considered excellent (Barker et al., 1994; Ansari et al., 2025).

Finally, hierarchical multiple regression analyses were conducted to examine the associations between the 11 AFML subscale scores and self-reported scores on life satisfaction, positive affect, negative affect, and perceived social support. Gender and musical training experience were entered at Step 1 as control variables. As the 11 AFML dimensions represent related but conceptually distinct music-listening functions, they were entered simultaneously at Step 2 to estimate the unique association of each function with the criterion variables while controlling for the variance shared with the other dimensions. The change in explained variance (Δ*R*^2^) at Step 2 represented the additional variance explained by the AFML dimensions beyond the control variables. Multicollinearity was assessed using tolerance and variance inflation factor (VIF) values.

## Results

### Construct validity

In the first stage, the hypothesized 11-factor model of the 46-item Chinese AFML was examined using data from Sample 1. The initial model showed acceptable fit, χ^2^ = 1462.932, χ^2^/*df* = 1.57, RMSEA = 0.020 (90% CI [0.018, 0.022]), CFI = 0.975, TLI = 0.972, and SRMR = 0.027. However, Item 37 (“When listening to music, I do not admire the talent of the performers”) showed a standardized factor loading of 0.317, which was below the prespecified criterion of 0.40. Based on statistical and theoretical considerations, Item 37 was removed. Specifically, its negative wording and relatively narrow focus on performers’ talent may have reduced its coherence with the broader Awe and Appreciation factor.

The revised 45-item, 11-factor model was then re-estimated using Sample 1. The model showed improved fit, χ^2^ = 1291.424, χ^2^/*df* = 1.45, RMSEA = 0.018 (90% CI [0.016, 0.020]), CFI = 0.980, TLI = 0.978, and SRMR = 0.020. These results supported the hypothesized 11-factor structure. As shown in Table 1, the standardized factor loadings of the retained items ranged from 0.435 to 0.927, all exceeding the prespecified criterion of 0.40 (Stevens, 2002).

**Table 1:** Standardized factor loadings of the AFML scale items in sample 1 and sample 2.

Dimension	Item	Standardized factor loadings	
		Sample 1	Sample 2
Stress regulation	1. Listening to music distracts me from stress	0.803	0.825
	2. When I feel stressed listening to music helps to take my mind off it	0.894	0.875
	3. I can escape from stressful situations by listening to music	0.903	0.899
	4. When I feel stressed I get comfort from listening to music	0.901	0.887
Strong emotional experiences	5. When listening to music I feel intense emotions	0.895	0.864
	6. When listening to music I feel a range of emotions	0.911	0.884
	7. When listening to music I feel emotions deeply	0.916	0.920
	8. When listening to music I feel a variety of emotions simultaneously	0.906	0.929
	9. When listening to music I feel a mixture of many different emotions	0.894	0.916
	10. I feel strong emotions when listening to music	0.857	0.885
Rumination	11. When I feel sad/depressed listening to music makes me dwell upon those feelings	0.885	0.913
	12. When I feel sad/depressed listening to music leads me to focus on those feelings	0.901	0.912
	13. When I feel anxious listening to music makes me dwell upon those feelings	0.892	0.910
	14. When I feel anxious listening to music leads me to focus on those feelings	0.896	0.889
Sleep	15. Listening to music in bed helps me fall asleep	0.916	0.913
	16. I listen to music in bed because it helps me get to sleep	0.886	0.921
Reminiscence	17. Listening to music does not bring back memories for me (R)	0.435	0.427
	18. When listening to music I reminisce about the past	0.883	0.891
	19. When listening to music I remember my past	0.880	0.916
	20. Listening to music reminds me of people from my past	0.870	0.898
Anger regulation	21. When I feel angry listening to music helps me look on the bright side	0.882	0.877
	22. When I feel angry listening to music helps me see things in a more positive light	0.899	0.891
	23. When I feel angry listening to music helps to take my mind off it	0.901	0.898
	24. When I feel angry listening to music distracts me from feelings of anger	0.910	0.906
	25. When I feel angry I listen to music that makes me happy	0.870	0.910
	26. When I feel angry listening to my favorite music makes me feel happier	0.889	0.902
	27. When I feel angry I get comfort from listening to music	0.891	0.908
Anxiety regulation	28. When I feel anxious listening to music helps me look on the bright side	0.892	0.883
	29. When I feel anxious listening to music helps me see things in a more positive light	0.914	0.906
	30. When I feel anxious listening to my favorite music makes me feel happier	0.892	0.914
	31. When I feel anxious I listen to music that makes me happy	0.919	0.916
	32. Listening to music distracts me from feelings of anxiety	0.914	0.908
	33. When I feel anxious listening to music helps to take my mind off it	0.892	0.909
	34. When I feel anxious I get comfort from listening to music	0.896	0.905
Awe and appreciation	35. Listening to music I feel a sense of awe for the talent of the composer	0.927	0.904
	36. Listening to music I feel a sense of awe for the talent of the performer	0.905	0.915
	37. When listening to music I do not admire the talent of the performers (R)		
Loneliness regulation	38. I feel less lonely when I listen to music	0.879	0.910
	39. Listening to music reduces feelings of loneliness	0.908	0.916
	40. Listening to music makes me feel less alone	0.927	0.924
Cognitive regulation	41. Playing music in the background helps me to concentrate	0.912	0.916
	42. Having background music makes it easier to focus on what I’m doing	0.896	0.900
Identity	43. Music listening is a fundamental part of who I am	0.890	0.892
	44. The music I listen to expresses who I am as a person	0.866	0.878
	45. Listening to music has helped me discover who I am	0.904	0.904
	46. Listening to music has helped me to understand myself	0.884	0.899

For independent cross-validation, the revised 45-item, 11-factor model was subsequently tested in Sample 2 (*n* = 1,415) without further modifications. The model also showed good fit, χ^2^ = 1291.706, χ^2^/df = 1.45, RMSEA = 0.018 (90% CI [0.016, 0.020]), CFI = 0.981, TLI = 0.979, and SRMR = 0.019. Standardized factor loadings ranged from 0.419 to 0.929, with all loadings exceeding 0.40. These findings further supported the stability of the revised 11-factor structure across the two independent subsamples.

### Measurement invariance

To examine whether the 11-factor model was invariant across gender and music training experience, a series of multigroup CFAs were conducted. First, the 11-factor model was tested separately within each group (i.e., women vs. men; less than 3 years vs. 3 years or more of music training). As shown in Table 2, configural, metric, and scalar invariance were then tested sequentially. For both gender and music training experience, the changes in fit indices between the configural and metric models (ΔCFI < 0.01 and ΔRMSEA < 0.01) supported metric invariance, whereas the changes between the metric and scalar models (ΔCFI ≤ 0.01 and ΔRMSEA < 0.01) supported scalar invariance. These results indicated that the AFML scale demonstrated scalar invariance across both gender and music training experience. Thus, comparisons of mean-level differences in AFML dimensions across gender and music training experience are meaningful.

**Table 2:** Measurement invariance tests of the Chinese AFML across gender and musical training.

Models	χ^2^	df	CFI	TLI	RMSEA(90% CI)	ΔCFI	ΔRMSEA
Gender							
Men	1145.459	890	0.986	0.984	0.015 (0.013, 0.018)	—	—
Women	1325.808	890	0.981	0.979	0.017 (0.015, 0.019)	—	—
Configural invariance	2473.701	1780	0.983	0.981	0.017 (0.015, 0.018)	—	—
Metric invariance	2547.350	1825	0.982	0.981	0.017 (0.015, 0.018)	0.001	0.000
Scalar invariance	2624.767	1870	0.982	0.981	0.017 (0.015, 0.018)	0.000	0.000
Musical training							
Less than 3 years	1316.394	890	0.984	0.983	0.015 (0.013, 0.017)	—	—
3 years or more	1286.522	890	0.971	0.967	0.024 (0.021, 0.027)	—	—
Configural invariance	2605.543	1780	0.980	0.978	0.018 (0.017, 0.020)	—	—
Metric invariance	2685.539	1825	0.980	0.978	0.018 (0.017, 0.020)	0.000	0.000
Scalar invariance	2762.902	1870	0.979	0.978	0.018 (0.017, 0.020)	0.001	0.000

### Gender and music training differences in AFML dimensions

Table 3 presents the means and standard deviations of the AFML dimensions by gender. Independent-samples *t*-tests revealed significant gender differences across all 11 AFML dimensions, with women scoring higher than men on each dimension (*t* > 3.39, *p* < 0.001, *d* > 0.13). Similarly, individuals with 3 years or more of music training scored significantly higher than those with less than 3 years of training on all AFML dimensions (*t* > 4.87, *p* < 0.001, *d* > 0.18).

**Table 3:** Gender and musical training differences in Chinese AFML dimensions.

AFML dimensions	Men		Women		*t*	*d*	Less than 3 years		3 years or more		*t*	*d*
	*M*	*SD*	*M*	*SD*			*M*	*SD*	*M*	*SD*		
1. Stress Regulation	3.39	0.87	3.60	0.76	−6.82^***^	0.26	3.46	0.76	3.67	0.93	−6.33^***^	0.24
2. Strong emotional experiences	3.40	0.85	3.60	0.74	−6.69^***^	0.25	3.46	0.75	3.67	0.90	−6.42^***^	0.24
3. Rumination	3.33	0.87	3.44	0.83	−3.44^***^	0.13	3.34	0.80	3.53	0.96	−5.48^***^	0.21
4. Sleep	3.38	0.91	3.52	0.83	−4.29^***^	0.16	3.41	0.82	3.59	0.98	−4.87^***^	0.18
5. Reminiscence	3.33	0.79	3.43	0.67	−3.49^***^	0.13	3.34	0.68	3.51	0.83	−5.52^***^	0.21
6. Anger regulation	3.40	0.84	3.57	0.74	−5.70^***^	0.21	3.44	0.73	3.65	0.89	−6.35^***^	0.24
7. Anxiety regulation	3.42	0.83	3.60	0.74	−6.14^***^	0.23	3.47	0.73	3.68	0.89	−6.57^***^	0.25
8. Awe and appreciation	3.44	0.86	3.63	0.77	−6.24^***^	0.23	3.48	0.76	3.73	0.92	−7.44^***^	0.28
9. Loneliness regulation	3.42	0.85	3.58	0.76	−5.22^***^	0.20	3.45	0.75	3.68	0.90	−6.85^***^	0.26
10. Cognitive regulation	3.43	0.85	3.58	0.77	−4.83^***^	0.18	3.45	0.76	3.67	0.91	−6.48^***^	0.25
11. Identity	3.43	0.82	3.54	0.76	−3.69^***^	0.14	3.43	0.74	3.67	0.89	−7.09^***^	0.27

*p < 0.05, **p < 0.01, ***p < 0.001.

### Internal consistency and reliability

Internal consistency was assessed using Cronbach’s α and split-half reliability. As shown in Table 4, the 11 dimensions of the AFML exhibited good to excellent reliability. Cronbach’s α coefficients ranged from 0.836 to 0.969, and split-half reliability coefficients ranged from 0.833 to 0.953. These findings indicate that the AFML has sound internal consistency across all dimensions.

**Table 4:** Reliability of the Chinese AFML in sample 1, sample 2, and the total sample.

AFML dimensions	Number of items	Sample 1 (*n* = 1,414)	Sample 2 (*n* = 1,415)	Total sample (*n* = 2,829)
Stress Regulation	4	0.929	0.927	0.928
Strong emotional experiences	6	0.961	0.962	0.961
Rumination	4	0.941	0.948	0.944
Sleep	2	0.896	0.912	0.905
Reminiscence	4	0.830	0.843	0.836
Anger regulation	7	0.964	0.967	0.966
Anxiety regulation	7	0.968	0.970	0.969
Awe and appreciation	2	0.913	0.905	0.909
Loneliness regulation	3	0.931	0.940	0.936
Cognitive regulation	2	0.900	0.904	0.902
Identity	4	0.936	0.940	0.938

### Criterion validity of the AFML dimensions

As shown in Table 5, hierarchical regression analyses were conducted to examine the criterion validity of the AFML after controlling for gender and musical training experience. The 11 AFML dimensions were entered simultaneously at the second step. Multicollinearity diagnostics showed that tolerance values ranged from 0.095 to 0.315 and VIF values ranged from 3.18 to 10.53. Anxiety Regulation showed the highest VIF (VIF = 10.53; tolerance = 0.095), indicating substantial overlap among some AFML dimensions. Regarding positive affect, strong emotional experiences, anger regulation, anxiety regulation, loneliness regulation, and identity emerged as significant positive predictors, whereas rumination was a significant negative predictor. Stress regulation, sleep, reminiscence, awe and appreciation, and cognitive regulation were not significantly associated with positive affect. For negative affect, stress regulation, strong emotional experiences, anxiety regulation, awe and appreciation, and cognitive regulation were negatively associated with negative affect. In contrast, rumination, reminiscence, loneliness regulation, and identity were positively associated with negative affect. Regarding life satisfaction, sleep, anger regulation, and identity positively predicted life satisfaction, whereas rumination showed a significant negative predictive effect. The remaining AFML dimensions were not significantly associated with life satisfaction. With respect to social support, anxiety regulation, loneliness regulation, and cognitive regulation emerged as significant positive predictors, while rumination was negatively associated with social support. Altogether, the 11 dimensions of the AFML accounted for 23.6% of the variance in positive affect, 8.0% of the variance in negative affect, 12.9% of the variance in satisfaction with life, and 26.8% of the variance in perceived social support, demonstrating adequate criterion validity.

**Table 5:** Hierarchical regression analyses examining the criterion-related validity of the Chinese AFML.

AFML dimensions	Positive affect				Negative affect				Satisfaction with life				Perceived Social support			
	Δ*R*^2^	*B*	*SE*	*β*	Δ*R*^2^	*B*	*SE*	*β*	Δ*R*^2^	*B*	*SE*	*β*	Δ*R*^2^	*B*	*SE*	*β*
Step1	0.033^***^				0.031^***^				0.028^***^				0.025^***^			
Gender		0.051	0.031	0.031		−0.320^***^	0.035	−0.169		0.201^***^	0.044	0.085		0.148^***^	0.028	0.099
Musical training		0.325^***^	0.035	0.175		0.174^***^	0.040	0.082		0.347^***^	0.049	0.131		0.182^***^	0.031	0.109
Step2	0.236^***^				0.080^***^				0.129^***^				0.268^***^			
1. Stress Regulation		−0.020	0.035	−0.020		−0.224^***^	0.044	−0.196		0.000	0.054	0.000		0.014	0.031	0.016
2. Strong emotional experiences		0.149^**^	0.044	0.145		−0.137^*^	0.055	−0.117		0.027	0.067	0.018		0.058	0.039	0.063
3. Rumination		−0.123^**^	0.028	−0.127		0.135^***^	0.035	0.122		−0.144^**^	0.043	−0.104		−0.074^**^	0.025	−0.085
4. Sleep		−0.005	0.027	−0.005		−0.025	0.034	−0.023		0.104^*^	0.042	0.077		0.027	0.024	0.032
5. Reminiscence		−0.024	0.037	−0.021		0.452^***^	0.046	0.350		−0.052	0.057	−0.032		−0.005	0.033	−0.005
6. Anger regulation		0.126^*^	0.050	0.121		−0.083	0.063	−0.070		0.238^**^	0.076	0.160		0.082	0.044	0.087
7. Anxiety regulation		0.128^*^	0.055	0.122		−0.167^*^	0.069	−0.139		0.037	0.084	0.025		0.132^**^	0.049	0.140
8. Awe and appreciation		0.036	0.039	0.036		−0.147^**^	0.049	−0.128		0.055	0.059	0.039		0.038	0.034	0.042
9. Loneliness regulation		0.085^*^	0.040	0.083		0.160^**^	0.050	0.137		0.061	0.061	0.042		0.113^**^	0.035	0.123
10. Cognitive regulation		0.058	0.039	0.057		−0.103^*^	0.049	−0.089		0.076	0.060	0.053		0.083^*^	0.035	0.091
11. Identity		0.107^**^	0.041	0.103		0.195^***^	0.051	0.164		0.133^*^	0.062	0.090		0.041	0.036	0.044

*p < 0.05, ^∗∗^*p* < 0.01, ^∗∗∗^*p <* 0.001.

## Discussion

The present study examined the reliability and validity of the Chinese version of the Adaptive Functions of Music Listening Scale (AFML). Results confirmed that the Chinese version of the AFML retained the original 11-factor structure among Chinese university students. Extending prior research, the scale demonstrated scalar measurement invariance across gender and musical training experience, and all subscales showed good to excellent internal consistency. Furthermore, the Chinese AFML dimensions exhibited the largely expected patterns of associations with positive affect, negative affect, life satisfaction, and perceived social support, supporting its satisfactory criterion validity. Taken together, these findings provide empirical evidence for the applicability of the Chinese version of the AFML as a measure of adaptive music-listening functions among Chinese university students.

### Factor structure of the Chinese AFML and measurement invariance

Consistent with findings from Western samples, the present study successfully replicated the 11-factor structure of the Adaptive Functions of Music Listening Scale (AFML; Groarke and Hogan, 2018), encompassing Stress Regulation, Anxiety Regulation, Anger Regulation, Loneliness Regulation, Rumination, Reminiscence, Strong Emotional Experiences, Awe and Appreciation, Cognitive Regulation, Identity, and Sleep. The replication of this structure suggests that the music-listening functions identified in the original Western study can also be meaningfully differentiated among Chinese university students. This provides preliminary support for the applicability of the AFML in the Chinese context, although direct cross-cultural equivalence remains to be tested. Future research should recruit matched Eastern and Western samples, and employ multigroup confirmatory factor analysis to rigorously test cross-cultural measurement invariance.

Scalar measurement invariance of the AFML was supported across gender and musical training groups, indicating that observed group differences in AFML scores are unlikely to be attributable to measurement bias. This finding provides a basis for meaningful comparisons of music-listening functions across these groups. Consistent with previous findings (Mas-Herrero et al., 2013; Wang et al., 2023), women scored higher than men on nearly all AFML dimensions. This pattern may reflect gender differences in emotional sensitivity and music use, as women may be more likely to engage with music for emotion regulation, social connection, and mood management (Wuttke-Linnemann et al., 2019). Similarly, students with 3 years or more of musical training scored higher than those with less than 3 years of training on most adaptive music-listening functions. Musical training may enhance auditory sensitivity, emotional responsiveness, and reflective engagement with music, enabling students to use music more actively for cognitive regulation, self-expression, and esthetic appreciation (Che et al., 2026; Woody and McPherson, 2010). It is worth noting that the effect sizes for gender and musical training differences were small (ds = 0.13–0.28). Thus, these findings reflect modest average group differences rather than substantial differences in music-listening functions. These differences should therefore be cautiously interpreted.

### Affective, social, and cognitive functions of music-listening and well-being

The criterion validity of the AFML was evaluated by examining its associations with positive and negative affect, and satisfaction with life. Moreover, we extended previous studies by testing the association between AFML dimensions and perceived social support. In terms of the affective functions, music can elicit diverse affective experiences, including positive and negative emotions, intense emotional states, and feelings of reminiscence (Groarke and Hogan, 2018). Consistent with a previous study (Groarke and Hogan, 2018), Strong Emotional Experiences were positively associated with positive affect. However, unlike the findings of a previous study (Groarke and Hogan, 2018), which reported a positive association with negative affect, the present study found a negative association. One possible explanation may be that Chinese university students’ relatively limited exposure to intensely negative music (e.g., rock and heavy metal; Wang et al., 2025). Strong Emotional Experiences may represent emotionally powerful but not distress-inducing experiences that help release or transform negative emotions. Moreover, consistent with previous findings (Chin and Rickard, 2012; Groarke and Hogan, 2018), Reminiscence was positively associated with negative affect. This association may reflect both the developmental stage of emerging adulthood and the transition to university life. Music-evoked memories may elicit negative emotions, particularly when they are associated with loss, regret, or unresolved experiences (Gibbs and Egermann, 2021; Mehl et al., 2025). For Chinese university students, academic pressure, separation from family, and changes in social relationships may heighten the salience of memories of high school, family, or past relationships (Wang et al., 2024), thereby contributing to greater negative affect.

Mood regulation is one of the most important functions of music listening (Chin and Rickard, 2012). Consistent with previous findings, regulatory functions were associated with positive affect, negative affect, and life satisfaction. Specifically, anger regulation and anxiety regulation were positively associated with positive affect, whereas stress regulation and anxiety regulation were negatively associated with negative affect. In addition, anger regulation was positively associated with life satisfaction. These findings were generally consistent with previous studies (Chin and Rickard, 2012; Groarke and Hogan, 2018). Music listening may support well-being not simply through general mood enhancement, but by helping listeners manage specific emotional challenges. Future studies would benefit from testing the associations between these regulatory functions and specific emotional problems, such as anxiety and anger. Despite these adaptive functions of music, consistent with previous findings, Rumination was positively associated with negative affect and negatively associated with positive affect, life satisfaction, and perceived social support. This pattern suggests that rumination-oriented music listening may reinforce negative emotions rather than facilitate recovery, thereby undermining both well-being and social bonding (Carlson et al., 2015).

With regard to the social function of music, Loneliness Regulation was positively associated with both positive affect and perceived social support, as expected. This suggests that using music to cope with loneliness may provide emotional comfort and a sense of social connection. However, contrary to our hypothesis, Loneliness Regulation was also positively associated with negative affect. One possible explanation is that students experiencing greater loneliness or emotional distress may be more likely to use music as a compensatory strategy. Although music listening may offer temporary relief, it may not necessarily improve underlying interpersonal difficulties. Moreover, in the present study, Identity was positively linked to positive and negative affect, as well as life satisfaction. This mixed pattern may be understood from a developmental perspective. University students are in a critical period of identity exploration (Arnett, 2000). Identity-related music use, such as expressing personal values and constructing a coherent sense of self, may enhance positive emotional experiences and contribute to greater life satisfaction (Groarke and Hogan, 2018). However, identity exploration is also accompanied by uncertainty, self-reflection, and emotional sensitivity.

Regarding the cognitive functions of music, the effects of Awe and Appreciation and Cognitive Regulation were investigated. As anticipated, cognitive regulation was found to be negatively associated with negative affect. This pattern may be due to the role of cognitive regulation in helping students organize their thoughts and maintain goal-directed engagement (Groarke and Hogan, 2018; Schultheiss et al., 2008). Moreover, Awe and Appreciation was negatively associated with negative affect, which was inconsistent with a previous study (Groarke and Hogan, 2018). This finding may reflect that awe and appreciation served a restorative function, helping Chinese students broaden attention and gain distance from distress. In addition, as expected, Sleep was positively associated with life satisfaction. This might be because sleep-related music listening promotes relaxation, improves perceived sleep quality, and supports daily recovery (Groarke and Hogan, 2018; Hu et al., 2023).

### Strengths, limitations, and future directions

While the present study benefits from a large Chinese sample and comprehensive analyses of the essential psychometric properties of the questionnaire, several limitations should be noted. First, the sample was drawn only from university students in one province of China; therefore, generalizability to other age groups, such as adolescents and older adults, or to other occupational groups should be interpreted with caution. Future research should examine measurement invariance and establish norms for the Chinese AFML across broader populations. Second, longitudinal studies are needed to further evaluate test–retest reliability and to investigate the dynamic mechanisms linking music-listening functions to well-being. Third, the traditional Chinese concept of “rites and music as complementary” highlights the role of music in moral cultivation and social harmony (Boer and Abubakar, 2014). Future studies would benefit from paying more attention to these culture-specific functions of music listening.

## Conclusion

This study translated and adapted the Adaptive Functions of Music Listening Scale into Chinese and systematically examined its psychometric properties among Chinese university students. The Chinese version of the AFML retained the original 11-factor structure and demonstrated good construct validity, criterion validity, internal consistency, and measurement invariance across gender and musical training experience. These findings support the Chinese AFML as a reliable and valid instrument for assessing the multidimensional functions of music listening in this population. Beyond its psychometric contribution, the present findings suggest that music listening may constitute an accessible resource for promoting well-being when used for adaptive purposes, such as emotion regulation, cognitive regulation, self-reflection, and social connection. However, the maladaptive patterns associated with rumination and reminiscence indicate that not all music-listening functions are beneficial. Students who become absorbed in music-evoked negative emotional states may benefit from guidance aimed at fostering more constructive listening habits. In addition, the mixed associations of identity-related music use with well-being suggest that music may amplify both positive and negative emotional experiences, highlighting the importance of considering the personal meaning and emotional context of music listening in university mental health services.

## Data Availability

The raw data supporting the conclusions of this article will be made available by the authors, without undue reservation.
